# Chronic spontaneous urticaria: Evidence of systemic microcirculatory changes

**DOI:** 10.1002/clt2.12335

**Published:** 2024-01-27

**Authors:** Yora Mostmans, Marcus Maurer, Bertrand Richert, Vanessa Smith, Karin Melsens, Viviane De Maertelaer, Ines Saidi, Francis Corazza, Olivier Michel

**Affiliations:** ^1^ Department of Immunology‐Allergology CHU Brugmann Université Libre de Bruxelles (ULB) Laken Belgium; ^2^ Department of Dermatology CHU Brugmann ULB Laken Belgium; ^3^ Institute of Allergology Charité–Universitätsmedizin Berlin Corporate Member of Freie Universität Berlin and Humboldt‐Universität zu Berlin Berlin Germany; ^4^ Fraunhofer Institute for Translational Medicine and Pharmacology ITMP Allergology and Immunology Berlin Germany; ^5^ Department of Internal Medicine Ghent University Ghent Belgium; ^6^ Department of Rheumatology Ghent University Hospital Ghent Belgium; ^7^ Unit for Molecular Immunology and Inflammation VIB Inflammation Research Center (IRC) Ghent Belgium; ^8^ Department of Rheumatology Vrije Universiteit Brussel (VUB) Universitair Ziekenhuis Brussel (UZ Brussel) Brussels Belgium; ^9^ IRIBHM Statistical Unit Fac. Medicine ULB Brussels Belgium; ^10^ Department of Immunology Laboratoire Hospitalier Universitaire de Bruxelles – Universitair Laboratorium Brussels (LHUB‐ULB) Brussels Belgium

**Keywords:** chronic spontaneous urticaria, ELISA, endothelial cell markers, microcirculation, videocapillaroscopy

## Abstract

**Background:**

Chronic spontaneous urticaria (CSU) is a chronic inflammatory skin disease where activation of endothelial cells (ECs) at sites of skin lesions leads to increased blood flow, leakage of fluid into the skin, cellular infiltration, and vascular remodeling. To understand the disease duration and the sometimes vague systemic symptoms accompanying flares, the objective of this study was to examine if CSU comes with systemic vascular changes at the microcirculatory level.

**Methods:**

We investigated CSU patients (*n* = 49) and healthy controls (HCs, *n* = 44) for microcirculatory differences by nailfold videocapillaroscopy (NVC) and for blood levels of the soluble EC biomarkers serum vascular endothelial growth factor (VEGF), soluble E‐selectin, and stem cell factor (SCF). Patients were also assessed for clinical characteristics, disease activity, and markers of autoimmune CSU (aiCSU).

**Results:**

CSU patients had significantly lower capillary density, more capillary malformations, and more irregular capillary dilations than HCs on NVC. Serum levels of VEGF, soluble E selectin and SCF were similar in CSU patients and HCs. CSU patients with higher VEGF levels had significantly more abnormal capillaries. Patients with markers of aiCSU, that is, low IgE levels or increased anti‐TPO levels, had significantly more capillaries and less capillary dilations than those without.

**Conclusion:**

Our results suggest that CSU comes with systemic microcirculatory changes, which may be driven, in part, by VEGF.

## 1 INTRODUCTION

Chronic spontaneous urticaria (CSU) is a chronic inflammatory skin disease characterized by the spontaneous activation of skin mast cells (MCs) with subsequent degranulation and release of histamine and other pro‐inflammatory mediators.^1^ Several pathomechanisms have been investigated in CSU, including infections, coagulation cascade, genetic factors, and autoimmunity.^2^ The latter is thought to be the most frequent underlying cause of CSU. Two types of hypersensitivity reactions, resulting in MC activation, have been postulated to be relevant in differentiating two autoimmune (ai)CSU endotypes^3^; in autoallergic (aa)CSU, also called type I autoimmune CSU (aiCSU), MC activation is driven by Immunoglobulin (Ig) E antibodies directed against autoantigens, that is, autoallergens, such as thyroid peroxidase (TPO). In Type IIb aiCSU, MCs are activated by IgG autoantibodies directed against IgE or its receptor FcεRI. Type I aiCSU is more common than type IIb aiCSU; the two endotypes overlap in some patients.^4^ Downstream of MC activation, in both endotypes, the endothelium of skin blood vessels becomes activated, resulting in vasodilation and extravasation, clinically visible as erythema and itchy wheals and/or angioedema (AE), respectively.^5^ These signs and symptoms of CSU develop repeatedly and resolve spontaneously. Every time new wheals develop, endothelial cells (ECs) become activated, resulting in increased blood flow, fluid leakage into the skin, cellular infiltration, and vascular remodeling.^6^ It is unknown whether activation of ECs is limited to sites of MC activation.

Nailfold videocapillaroscopy (NVC) is used to study microcirculatory features, including capillary density, dimension, and morphology. NVC is non‐invasive, can detect minimal microcirculatory changes, and is used in diagnosing patients with rheumatic diseases that come with microangiopathies.^7^, ^8^ Reduced NVC counts of capillaries point to systemic endothelial damage. NVC findings of capillary dilation and abnormalities, with tortuous, enlarged and/or disarranged/ramified capillaries, reflect neo‐angiogenesis. Several autoimmune diseases (AID), including systemic sclerosis and systemic lupus, have lower capillary densities and capillary abnormalities as seen via NVC.^9^, ^10^ It is currently unknown whether CSU, which is autoimmune in a subpopulation of patients, accompanies systemic microcirculatory changes.

In CSU, the activation of skin ECs in response to MC degranulation involves the effects of histamine and other mediators (which may include Vascular Endothelial Growth Factor [VEGF])^11^ on H1 receptors.^12^ VEGF is a potent regulator of angiogenesis, and its expression is abundant in several chronic inflammatory diseases. VEGF induces proliferation, migration and tube formation of ECs and promotes the expression of chemokines as well as leukocyte adhesion molecules, such as E‐selectin. VEGF is produced and secreted by MCs including human skin MCs,^13^, ^14^ and the serum of patients with CSU can induce MCs to secrete VEGF in vitro.^15^ Also, increased blood and lesional skin levels of VEGF have been reported in CSU patients,^16^, ^17^, ^18^ although this has not been confirmed.^19^, ^20^

E‐selectin is a cell adhesion molecule constitutively expressed in skin and bone marrow microvessels and in other tissues strongly upregulated by inflammatory cytokines, such as tumor necrosis factor‐*α* and interleukin‐1*β*.^21^, ^22^, ^23^ It plays an important role in transendothelial cell migration,^6^, ^24^ and is upregulated in non‐lesional and lesional skin of urticaria patients.^11^

There is also evidence of vascular remodeling in CSU skin, with significantly more CD31+ ECs in lesional versus non‐lesional skin.^25^ Patients with CSU with high numbers of cutaneous CD31+ ECs have high rates of recurrent AE.^26^ Not only the formation of new blood vessels, but also the formation of blood cells (hematopoiesis), f.e. through Stem Cell Factor (SCF), has been examined in CSU. Activated ECs release SCF, which is commonly known as the ligand for c‐KIT receptor, the main growth factor of MCs.^27^, ^28^ Nevertheless, the role of SCF in CSU pathogenesis remains insufficiently understood.^29^, ^30^

Since the integrity of vessel walls is compromised in recurring CSU wheals, we examined if there are changes in the microcirculation outside of the lesional skin area. Therefore, the objectives of this study were to firstly identify whether systemic vascular changes in CSU occur, and secondly, to detect any differences between CSU endotypes (clinically through NVC and biologically through the assessment of specific soluble EC biomarkers [VEGF, sE‐selectin, and SCF]).

## 2 MATERIAL AND METHODS

### 2.1 Study subjects and design

This cross‐sectional, cohort study enrolled 49 adult patients with CSU through written informed consent at the Immunology‐Allergology Department of the Center Hospitalier Universitaire (CHU) Brugmann, Université Libre de Bruxelles (ULB), Brussels, Belgium, between October 2018 and September 2021. Most patients (67%) were female, the average age was 38.6 ± 2.1 years, and 73% were <45 years old. All patients had expert‐confirmed CSU, with recurrent wheals (hives) with or without AE for longer than 6 weeks.^31^ Patients with standalone chronic inducible urticaria, AE only, or wheals due to other causes, that is, infections or food/drug cross‐intolerance, were excluded based on clinical history and appropriate investigations. Inclusion criteria consisted of: (a) age >17 years, (b) no antihistamine intake during the 7 days prior to inclusion, (c) not using omalizumab, corticosteroid drugs or cyclosporin treatment at the time of inclusion, (d) no anti‐inflammatory drugs at the time of inclusion, and (e) no history of systemic connective tissue disorders. Study patients were assessed for CSU symptom onset, frequency and activity level, diurnal variation, general CSU symptoms and accompanying gastro‐intestinal complaints, CSU triggers, previous CSU treatment, occurrence and characteristics of AE, cardiovascular profile, and history of mental health issues. Also, presence of stress, personal and familial medical history were documented, based on the subjective assessment given by the patient.

Furthermore, 44 age‐ and sex‐matched healthy controls (HCs) were included in the study (64% female, mean age: 37.7 ± 2.0 years), 24 at the CHU Brugmann hospital and 20 at the Rheumatology department of the University Hospital in Ghent (Universitair Ziekenhuis [UZ] Ghent), Belgium, during the same period (October 2018–September 2021).

### 2.2 Patient‐reported outcome measures and comorbidity risk stratification tools

CSU disease activity was determined using the weekly Urticaria Activity Score (UAS7) and defined as: urticaria‐free (0 points); well‐controlled/minimal disease activity (1–6 points); mild disease activity (7–15 points); moderate disease activity (16–27 points); and severe disease activity (28–42 points).^32^ SCORE2 (Systematic Coronary Risk Evaluation) and SCORE2‐Older Persons (SCORE2‐OP; for patients aged >69 years) risk prediction algorithms were used to estimate if the 10‐year risk of cardiovascular events was low (Grade 1), moderate (Grade 2), or high (Grade 3).^33^, ^34^ Patients were categorized as obese when their body mass index score was ≥30 kg/m^2^.^35^

Patients with CSU were assessed for markers of type IIb aiCSU, that is, history or presence of AID, antinuclear antibodies (ANAs) and low IgE levels and elevated anti‐TPO levels. As for the latter two, patients were categorized into two groups: (a) patients with low IgE levels, that is, <40 IU/mL, and/or increased anti‐TPO levels (*n* = 19)^36^ and (b) patients with high/normal IgE levels and normal anti‐TPO levels (*n* = 29). Furthermore, NVC parameters were compared in patients with low IgE values versus normal/high IgE values and in patients with increased anti‐TPO levels versus normal anti‐TPO levels.

### 2.3 Autologous serum skin tests

The autologous serum skin test, ASST is a test for autoreactivity that evaluates the presence of serum histamine‐releasing factors including histamine‐releasing autoantibodies. The ASST was performed according to the European Academy of Allergy & Clinical Immunology/GA^2^LEN task force consensus procedure as previously described.^37^ Wheal responses were measured at 15 and 30 min after the intradermal injection of 50 μL of autologous serum. When a red serum‐induced wheal had a diameter of at least 1.5 mm greater than the negative control, the ASST response was considered positive.

### 2.4 Blood analysis

Venous blood was collected from patients with CSU (on the same day as the NVC imaging) and from a part of the HC group (due to logistical reasons, the HCs from UZ Ghent did not participate in this sub study). VEGF, E‐selectin, and SCF plasma concentrations were determined using enzyme‐linked immunosorbent assay (ELISA), in accordance with the manufacturer's instructions (R&D Systems Inc., Minneapolis, MN, USA).

### 2.5 Nailfold videocapillaroscopy

CSU and HC study subjects were also investigated by standardized NVC as described by Smith et al^7^. All fingers, except for the thumbs, were assessed with a x200 magnification contact lens (Optilia microscope). Two images per nailfold/finger were captured, coded, and saved (sets of 16 images per patient).

The NVC images were randomized, read, and reported by a trained European League Against Rheumatism (EULAR)‐certified capillaroscopic expert (YM) blinded to the subject's health or disease status. We followed the capillaroscopic protocol from the EULAR study group on microcirculation in Rheumatic Diseases (SG MC/RD) definitions that is, the quantitative NVC‐assessment in a 1 mm grid^8^, ^38^:

**Table:** 

Capillary density	The mean number of capillaries in the distal row per linear mm
Capillary dimension	The mean number of dilated capillaries (dilations) when the apical limb diameter was 20–50 μm and the mean number of giant capillaries when the apical limb diameter was >50 μm; where regular dilations were defined as homogeneously enlarged capillaries and irregular dilations were considered when a segment of the vessel was more dilated than the rest; with the number of dilations, relative to the total number of capillaries per linear mm, represented as percentage, that is, relative capillary dimension
Capillary morphology	The mean number of capillaries with a normal morphology: Capillaries with a hairpin shape, once or twice‐crossing shape, tortuous shape that is, limbs bend but not crossed; on the condition that the tip of the capillary is convex
	Abnormal morphology: All capillaries whose shape does not correspond to the definition of a normal shape, subcategorized as ramified, concave tip or > twice‐crossing shape^7^, ^10^
	The number of abnormally shaped capillaries in relation to the sum of all, that is, relative capillary morphology
Microhemorrhages	Mean number of red or brown amorphous structures in the pericapillary and/or periungual region

For descriptive purposes, the subpapillary venous plexus visibility score was also graded from 0 to 3 (0: not visible; 1: doubtful visibility; 2: plexus visible only in restricted areas; 3: prominently visible over a wide area).^9^, ^39^

### 2.6 Statistics

Continuous variables are summarized as means and their standard deviation (SD), and qualitative variables as numbers and percentages. Differences in the means of continuous variables between two independent groups were compared using classical Student *t*‐tests or Welch's *t*‐tests in case of variance heterogeneity. Associations between each considered continuous variables and the other variables of interest were investigated using multiple regressions analyses, including age as a covariate and sex as a factor. More than two independent groups were compared using analyses of variance (ANOVA) followed by Sidak or Dunnett T3 multiple comparison tests when required according to the results of the Levene test for homoscedasticity. Differences between groups and associations concerning qualitative variables were analyzed using Pearson's exact chi‐square tests. Statistical significance was considered when *p* was <0.05. All statistical tests were two‐sided and performed using IBM‐SPSS (version 28.0) software.

## 3 RESULTS

### 3.1 Demographics, clinical characteristics, and laboratory results of patients with chronic spontaneous urticaria

Most patients (70%) had moderate or severe disease activity, based on UAS7 values, 80% had wheals every day, and 49% had CSU for more than 1 year (Table 1). Across all patients, 71% reported more night‐time than daytime whealing. AE occurred in 67% of patients, and 78% with AE had attacks at least once per month. In 67% of patients with AE, the lower lip was affected, and 9% reported genital AE. One in five patients (22%), previously or currently, had an autoimmune comorbidity, most commonly Hashimoto's thyroiditis (36%).

**TABLE 1:** Clinical characteristics of CSU patients.

Clinical characteristics	CSU (*n* = 49)
Age, *n* (%)	
≤45years	36/49 (73.5)
>45years	13/49 (26.5)
Sex, n (%)	
Male	16/49 (32.7)
Female	33/49 (67.4)
Disease activity level, *n* (%)^†^	
urticaria‐free	0/46 (0)
well‐Controlled/minimal disease activity	2/46 (4.4)
mild disease activity	12/46 (26.1)
moderate disease activity	17/46 (37)
severe disease activity	15/46 (32.6)
Disease duration, *n* (%)	
<3m	4/49 (8.2)
<6m	11/49 (22.5)
<1year	10/49 (20.4)
>1year	24/49 (50)
Disease frequency, *n* (%)	
Every day	39/49 (79.6)
3x/week	9/49 (18.4)
Every week	0/49 (0)
Every month	1/49 (2)
Diurnal variation, *n* (%)	
No variation	10/49 (20.4)
More at day	4/49 (8.2)
More at night	35/49 (71.4)
Accompanying AE, *n* (%)	
No	16/49 (32.7)
Yes	33/49 (67.4)
Frequency^†^	
every day	2/32 (6.3)
3x/week	4/32 (12.5)
1x/week	10/32 (31.3)
every month	10/32 (31.3)
2‐3x/y	6/32 (18.8)
Location	
upper lip	21/33 (63.6)
lower lip	22/33 (66.7)
upper eyelid R	15/33 (45.5)
upper eyelid L	18/33 (54.6)
lower eyelid R	15/33 (45.5)
lower eyelid L	13/33 (39.4)
genital region	3/33 (9.1)
Tongue	5/33 (15.2)
CSU symptoms, *n* (%)	
Itch	49/49 (100)
Burn	38/49 (77.6)
Tight skin	23/49 (46.9)
Pain	17/49 (34.7)
Joint dysfunction	12/49 (24.5)
History, *n* (%)	
Allergy in personal history	31/49 (63.3)
Allergy in the family^†^	27/48 (56.3)
*H pylori* gastritis	2/49 (4.1)
CV profile, *n* (%)	
Obesity	18/49 (36.7)
Arterial Hypertension	14/49 (28.6)
Hypercholesterolemia	13/49 (26.5)
Smoking	9/49 (18.4)
SCORE2 and score2‐OP risk	
Grade 0	27/49 (55.1)
Grade 1	8/49 (16.3)
Grade 2	12/49 (24.5)
Grade 3	2/49 (4.1)
History of AID, *n* (%)	11/49 (22.5)
Hashimoto's disease	4/11 (36.4)
AI gastritis	2/11 (18.2)
Alopecia areata	2/11 (18.2)
Vitiligo	2/11 (18.2)
Rheumatoid arthritis	1/11 (9.1)
Diabetes type 1	1/11 (9.1)
Psoriasis	1/11 (9.1)
Graves‐Basedow thyroiditis	1/11 (9.1)
Psychiatric/psychologic history, *n* (%)	13/49 (26.5)
Depression	9/13 (69.2)
Anxiety	3/13 (23.1)
Burn out	2/13 (15.4)
ADHD	1/13 (7.7)
Aggression	1/13 (7.7)
Gastro‐intestinal complaints, *n* (%)	29/49 (59.2)
Pyrosis	18/29 (62.1)
Constipation	8/29 (27.6)
Diarrhea	7/29 (24.1)
Flatulence	7/29 (24.1)
CSU triggers, *n* (%)	40/49 (81.6)
Friction	30/40 (75)
Heat	22/40 (55)
Exercise	13/40 (32.5)
Stress	8/40 (20)
Physical agents	7/40 (17.5)
Vibration	5/40 (12.5)
Sun	5/40 (12.5)
Cold	3/40 (7.5)
Pressure	2/40 (5)
Changes in temperature	2/40 (5)
Other	2/40 (5)
Stress, *n* (%)	
No	6/49 (12.2)
Mild	8/49 (16.3)
Moderate	17/49 (34.7)
Severe	18/49 (36.7)
Cause of stress^†^	
Family issues	28/43 (65.1)
Work/school	26/43 (60.5)
Disease	26/43 (60.5)
Other	2/43 (4.7)
Previous CSU treatment, *n* (%)	
No	1/49 (2)
AH	47/49 (95.9)
Oral CS	11/49 (22.5)
Omalizumab	0/49 (0)
Cyclosporin	0/49 (0)
Dapsone	0/49 (0)
Other	2/49 (4.1)

*Note*: Clinical data of patients with CSU are given as absolute numbers and percentages calculated from the data available (mostly *n* = 49, except for ^†^ where there was missing data in the patient's medical charts).

Abbreviations: ADHD, attention deficit hyperactivity disorder; AE, angioedema; AH, antihistamines; AI, auto‐immune; AID, auto‐immune diseases; CS, corticosteroids; CSU, chronic spontaneous urticaria; CV, cardiovascular.

One in three patients (35%) had elevated C‐reactive protein levels, and one in four (26%) had increased erythrocyte sedimentation rate levels (Table 2). IgE levels were low (<40 IU/ml) and high (>100 IU/ml) in 25% and 50% of patients, respectively, and one fifth of patients (19%) had circulating anti‐TPO antibodies. IgE levels were low and/or anti‐TPO levels were high in 19/49 (39%) patients. This patient group did not significantly differ in disease duration nor disease activity from patients with high/normal IgE levels and/or normal anti‐TPO levels (*p* = 0.51 and *p* = 0.88 respectively).

**TABLE 2:** Serologic findings of CSU patients and controls.

Serologic parameters, reference, mean (SD)	CSU (*n* = 48)	HC (*n* = 20)	*p*‐value
Bloodcount			
Leukocytes, 3.5–11 × 10^3^/μL	4.80 (0.07)	4.86 (0.12)	0.56
Erythrocytes, 4.4–5.9 × 10^6^/μL	7.14 (0.25)	7.42 (0.43)	ND
Platelets, 150‐440 × 10^3^/μL	288.21 (10.08)	270.65 (15.11)	0.34
Inflammation			
ESR, <15 mm/h	15.04 (1.92)^1^	14.85 (2.38)	0.95
CRP, <5 mg/L	6.43 (1.14)	3.61 (1.13)	0.15
Renal function			
Creatinine, 0.7–1.2 g/L	0.84 (0.02)	0.82 (0.03)	ND
Hepatic function			
Total bilirubin, <1.2 mg/dL	0.51 (0.06)	0.38 (0.06)	ND
Direct bilirubin, <0.2 mg/dL	0.19 (0.02)	0.18 (0.02)	ND
ALT, <41 UI/L	21.15 (1.87)	17.85 (1.58)	ND
AST, <40 UI/L	20.60 (0.91)	19.50 (1.05)	ND
GGT, 10–71 UI/L	22.77 (2.0)	28.70 (6.9)	ND
ALP, 40–129 UI/L	73.02 (2.86)	68.60 (4.52)	ND
Thyroid function			
TSH, 0.27–4.20 mU/L	2.05 (0.16)	1.54 (0.19)	0.08
fT4, 12–22 pmol/L	15.66 (0.42)	16.93 (0.73)	0.12
Anti‐TPO, <35 kUI/L	54.71 (15.83)	14.38 (1.11)^5^	**0.01**
Allergy			
IgE, 40–100 kU/L	164.02 (28.84)	159.35 (106.50)^5^	0.95
Tryptase, <11 μg/L	5.83 (0.33)	5.32 (0.67)	0.44
Complement			
C3, 0.9–1.8 g/L	1.26 (0.04)^1^	1.11 (0.06)	0.06
C4, 0.1–0.4 g/L	0.27 (0.01)^1^	0.25 (0.01)^5^	0.4
C3d, <1.1 mg/dL	0.90 (0.09)^2^	0.83 (0.14)	ND
C1q, 100–250 mg/L	192.77 (5.89)^3^	172.80 (6.55)	**0.04**
CH50, 41–94 U/mL	67.90 (2.63)^4^	65.18 (5.0)^5^	ND
Auto‐immunity (Abs)			
Presence of ANA, %	26.1^2^	10.5^5^	0.2
*H. Pylori* Ab,≥22 U/mL, %	39.5^3^	ND	ND

*Note*: Venous blood was collected from patients with CSU (*n* = 48) and from some of the HCs (*n* = 20). Mean data with standard deviations for different parameters are given. For some parameters, limited data was missing (^1^ *n* = 47; ^2^ *n* = 46; ^3^ *n* = 43; ^4^ *n* = 44; ^5^ *n* = 19). Tryptase was measured with an Immunocap Tryptase Phadia with a limit of quantification at 1 μg/L (measuring range: 1–200 μg/L). Levels below 11.4 μg/L were considered normal according to the manufacturer's cut‐off. *p*‐values <0.05 were considerate statistically significant (bold).

Abbreviations: Abs, antibodies; ALP, alkaline phosphatase; ALT, alanine transaminase; ANA, antinuclear antibodies; Anti‐TPO, anti‐thyroid peroxidase; AST, aspartate aminotransferase; C, complement; CH50, total complement activity; CRP, C‐reactive protein; CSU, chronic spontaneous urticaria; ESR, erythrocyte sedimentation rate; fT4, free thyroxine; GGT, gamma‐glutamyl transferase; HC, Healthy Controls; *H. Pylori*, *Helicobacter Pylori*; IgE, Immunoglobulin E; NA, not applicable; ND, not determined; NS, no significance; TSH, thyroid stimulating hormone.

### 3.2 Patients with chronic spontaneous urticaria have significantly lower capillary density, more irregular capillary dilations, and more abnormally shaped capillaries compared with healthy controls

**FIGURE 1:**
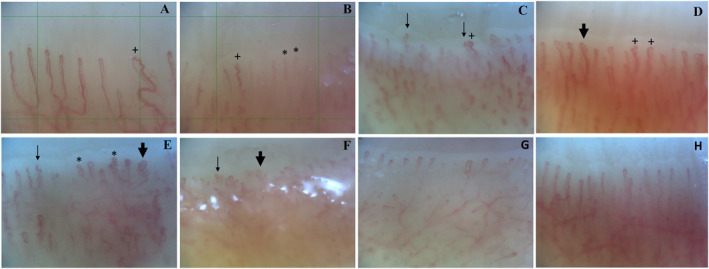
Microvascular abnormalities on NVC in patients with chronic spontaneous urticaria and healthy controls. NVC images of six different CSU patients (A–F) showing (A–B) decreased capillary density, 5 capillaries/1 mm grid and 6 capillaries/1 mm grid respectively, (A–D) irregular dilations (>20 μm) (+), and (B–F) abnormally shaped capillaries including multiple crossing capillaries (*), ramified (“bushy”) capillaries (bold arrow) and concave tips (regular arrow). NVC images of 2 different healthy controls (G–H) show a capillary density of >7/mm, with normally shaped hairpin capillary loops and capillary dimensions <20 μm. CSU, chronic spontaneous urticaria; NVC, nailfold videocapillaroscopy.

Patients with CSU and HCs exhibited stark differences in capillary density, dimensions, and morphology, as assessed by NVC: patients had significantly fewer capillaries (7.45 ± 0.87/mm vs. 8.50 ± 0.85/mm) and more capillaries with irregular dilations (0.09 ± 0.17/mm vs. 0.003 ± 0.01/mm) compared with HCs (both *p* < 0.001). Regarding capillary morphology, patients with CSU had markedly more abnormally shaped capillaries (0.62 ± 0.39/mm vs. 0.17 ± 0.16/mm). Specifically, they had more abnormally crossed capillaries (0.30 ± 0.28/mm vs. 0.05 ± 0.07/mm), ramified capillaries (0.17 ± 0.16/mm vs. 0.06 ± 0.10/mm), and capillaries with concave tips (0.15 ± 0.12/mm vs. 0.06 ± 0.10/mm) compared with HCs (all *p* < 0.001, Table 3, Figure 1). In contrast, patients with CSU and HCs did not differ in the presence of microhemorrhages (*p* = 0.38) and subpapillary venous plexus visibility scores (*p* = 0.67).

**TABLE 3:** ELISA results and explorative analysis of quantitative nailfold videocapillaroscopy parameters in CSU patients compared to controls.

Demographic data of included population	CSU (*n* = 49)	HC (*n* = 44)
Age, *n* (%)		
<35years	24/49 (49)	23/44 (52.3)
≥35years	25/49 (51)	21/44 (47.7)
Sex, *n* (%)		
Male	16/49 (32.7)	16/44 (36.4)
Female	33/49 (67.4)	28/44 (63.6)
Number of subjects with ELISA, n (%)	41/49 (83.7)	20/44 (45.5)
Number of subjects with NVC examination, n (%)	49/49 (100)	24/44 (54.6)

ELISA, mean values (pg/mL)	CSU (*n* = 41)	HC (*n* = 20)	*p*‐value
VEGF	527.55	483.46	0.61
E‐selectin	48.25	43.28	0.30
SCF	846.62	862	0.75

NVC quantitative parameters	CSU (*n* = 49)	HC (*n* = 24)	*p*‐value
Density			
Capillary density/mm, average (SD)	7.45 (0.87)	8.50 (0.85)	**<0.001**
Dimensions			
Nb of dilations/mm, average (SD)	0.60 (0.64)	0.40 (0.39)	0.16
Nb of regular dilations/mm, average (SD)	0.51 (0.54)	0.40 (0.39)	0.35
Nb of irregular dilations/mm, average (SD)	0.09 (0.17)	0.003 (0.01)	**<0.001**
Nb of dilations/total nb of capillaries per mm, average % (SD)	8.30 (9.29)	4.95 (5.16)	0.11
Morphology			
Nb of abnormal shapes/mm, average (SD)	0.62 (0.39)	0.17 (0.16)	**< 0.001**
Nb of crossings/mm, average (SD)	0.30 (0.28)	0.05 (0.07)	**< 0.001**
Nb of ramifications/mm, average (SD)	0.17 (0.16)	0.06 (0.10)	**< 0.001**
Nb of concave tip/mm, average (SD)	0.15 (0.12)	0.06 (0.10)	**< 0.001**
Microhemorrhages			
Nb of microhemorrhages, average (SD)	0.04 (0.08)	0.02 (0.07)	0.38
Subpapillary venous plexus			
Visibility score, average (SD)	0.37 (0.49)	0.42 (0.41)	0.67

*Note*: NVC images were taken at 200X magnification. At the subject level, the mean of each quantitative parameter was calculated. The averages of the means are reported in this table. Student *T*‐test was used for continuous variables with a cut‐off of *p* < 0.05 for statistical significance (bold). All data are averages ± SD for continuous variables. Standard definitions of capillaroscopic evaluations suggested by the European League Against Rheumatism study group on microcirculation in rheumatic diseases (EULAR SG MC/RD) were applied^7^, ^38^. NVC parameters were corrected for age and sex. Capillary crossings were considered abnormal when >2 crossings were observed. The total number of abnormal shapes corresponded with the sum of all capillaries with multiple crossings (>2), ramifications and concave tips.

Abbreviations: CSU, chronic spontaneous urticaria; ELISA, enzyme linked immunosorbent assay; F, female; HC, Healthy Controls; M, male; Nb, number; NVC, nailfold videocapillaroscopy; PCR, polymerase chain reaction; SCF, stem cell factor; SD, standard deviation; VEGF, vascular endothelial growth factor.

### 3.3 In chronic spontaneous urticaria, a history of autoimmune disease and/or low IgE and/or elevated anti‐thyroid peroxidase are linked to distinct microcirculatory alterations

Eleven of 49 patients with CSU (22%) with a previous or current history of AID (most commonly Hashimoto's thyroiditis) had less dilated capillaries than those without (0.27 ± 0.24/mm vs. 0.70 ± 0.69/mm, respectively, *p* = 0.048). Furthermore, patients with a history of AID had less regularly enlarged capillaries (0.23 ± 0.23/mm vs. 0.60 ± 0.58/mm, respectively; *p* = 0.049) compared with those without AID. Also, patients with CSU and a history of AID had more ramified capillaries (in absolute count/mm: 0.26 ± 0.2/mm vs. 0.14 ± 0.14/mm, *p* = 0.047) on NVC compared with CSU patients without a previous history of AID.

Patients with CSU with low IgE levels and/or increased anti‐TPO levels (19/48, 40%) had higher capillary density (7.75 ± 0.21/mm vs. 7.25 ± 0.15/mm, *p* = 0.01) and less dilated capillaries (0.32 ± 0.09/mm vs. 0.79 ± 0.13/mm, *p* = 0.003) on NVC compared with patients with normal/high IgE levels and/or normal anti‐TPO levels (29/48, 60%). In contrast, low IgE levels or elevated anti‐TPO alone, elevated levels of ANA, and positive ASST results were not linked to significant differences in NVC parameters (Table 4).

**TABLE 4:** Nailfold videocapillaroscopy findings in CSU patients with aiCSU characteristics compared to CSU patients without.

NVC quantitative assessment	Presence/History of AID (*n* = 11)	High Anti‐TPO (*n* = 9)	Low IgE (*n* = 12)	Low IgE and/or high anti‐TPO (*n* = 19)	Presence of ANA (*n* = 12)	Positive ASST (*n* = 26)
Mean capillary density/linear mm, (SD)	7.58 (0.99)	7.63 (0.32)	7.85 (0.27)	7.75 (0.21)	7.55 (0.27)	7.35 (0.83)
*p*‐value	0.87	0.24	0.06	**0.01**	0.83	0.72
Mean total nb of normal cap/linear mm, (SD)	6.89 (1.11)	ND	ND	7.03 (0.23)	ND	6.79 (0.95)
*p*‐value	0.97			0.14		0.91
Mean total nb of abnormal cap/linear mm, (SD)	0.70 (0.51)	0.72 (0.20)	0.65 (0.08)	0.72 (0.1)	0.62 (0.12)	0.55 (0.36)
*p*‐value	0.66	0.25	0.81	0.11	0.84	0.33
%Abnormal/total nb cap, (SD)	9.24 (0.26)	ND	ND	9.40 (1.42)	ND	7.67 (5.5)
*p*‐value	0.63			0.09		0.51
Mean total nb of ramified cap/linear mm, (SD)	0.26 (0.20)	0.22 (0.07)	0.17 (0.05)	0.21 (0.04)	0.20 (0.06)	0.15 (0.17)
*p*‐value	**0.047**	0.28	0.1	0.69	0.42	0.63
%Ramified/total nb cap, (SD)	42.45 (23.84)	ND	ND	29.58 (5.39)	ND	27.36 (22.35)
*p*‐value	**0.005**			0.98		0.55
Mean total nb of concave cap/linear mm, (SD)	0.18 (0.15)	0.16 (0.05)	0.18 (0.03)	0.18 (0.03)	0.18 (0.03)	0.14 (0.12)
*p*‐value	0.41	0.74	0.27	0.78	0.36	0.72
%Concave/total nb cap, (SD)	23.40 (13.36)	ND	ND	31.44 (5.78)	ND	33.80 (28.77)
*p*‐value	0.39			0.54		0.59
Mean total nb of crossing cap/linear mm, (SD)	0.25 (0.24)	0.34 (0.11)	0.30 (0.07)	0.32 (0.06)	0.23 (0.07)	0.26 (0.27)
*p*‐value	0.33	0.4	0.87	0.07	0.2	0.36
%Crossing/total nb cap, (SD)	34.14 (20.05)	ND	ND	39.91 (6.51)	ND	40.57 (32)
*p*‐value	0.18			0.44		0.91
Mean total nb of reg. enlarged cap/linear mm, (SD)	0.23 (0.23)	0.19 (0.03)	0.34 (0.13)	0.27 (0.08)	ND	0.57 (0.52)
*p*‐value	**0.049**	0.15	0.28	**0.01**		0.71
Mean total nb of irreg. enlarged cap/linear mm, (SD)	0.04 (0.06)	0.05 (0.03)	0.04 (0.02)	0.05 (0.02)	ND	0.10 (0.20)
*p*‐value	0.31	0.89	0.28	**0.006**		0.71
Mean total nb of giant cap/linear mm, (SD)	0 (0)	ND	ND	ND	ND	0 (0)
*p*‐value	0.69					0.19
Mean total nb of dilated cap/linear mm, (SD)	0.27 (0.24)	0.24 (0.05)	0.37 (0.14)	0.32 (0.09)	0.39 (0.17)	0.70 (0.66)
*p*‐value	**0.048**	0.2	0.22	**0.003**	0.24	0.68
%nb dil/tot nb cap, (SD)	3.68 (3.40)	ND	ND	3.9 (1.05)	ND	9.36 (9.40)
*p*‐value	0.07			**<0.001**		0.73
Mean total nb of microhemorrhages/linear mm, (SD)	0.01 (0.03)	0 (0)	0.03 (0.02)	0.02 (0.01)	0.05 (0.03)	0.04 (0.09)
*p*‐value	0.22	0.24	0.92	0.14	0.32	0.79
Mean NVC plexus score, (SD)	0.41 (0.36)	0.31 (0.10)	0.28 (0.10)	0.28 (0.07)	0.35 (0.10)	0.34 (0.53)
*p*‐value	0.67	0.76	0.51	0.49	0.99	0.49

*Note*: *P*‐values are considered statistically significant when *p* < 0.05 (bold).

Abbreviations: AID, auto‐immune diseases; ANA, antinuclear antibodies; ASST, autologeous serum skin test; CSU, chronic spontaneous urticaria; ELISA, enzyme linked immunosorbent assay; F, female; HC, Healthy Controls; IgE, Immunoglobulin E; irreg., irregularly; M, male; Nb, number; NVC, nailfold videocapillaroscopy; PCR, polymerase chain reaction; reg., regularly; SCF, stem cell factor; SD, standard deviation; TPO, thyroid peroxidase; VEGF, vascular endothelial growth factor.

### 3.4 Vascular endothelial growth factor, sE‐selectin and history of autoimmune disease were associated with changes in the microcirculation

VEGF and sE‐selectin blood levels in patients with CSU, were linked to microcirculatory changes detected by NVC, albeit similar to the levels in HCs (Table 5). Patients with high VEGF levels had significantly higher rates of abnormal capillaries (*p* = 0.02 for mean total abnormal cap and *p* = 0.004 for %abnormal/total nb cap, respectively), than those with low VEGF, especially more abnormal capillaries with >2 crossings (*p* = 0.02). Patients with high versus low sE‐selectin levels had fewer capillary dilations (*p* = 0.03) and irregular dilations (*p* = 0.04).

**TABLE 5:** Nailfold videocapillaroscopy characteristics in CSU patients and their clinico‐serological associations.

NVC parameters (*p*‐value)	SCORE2 (OPT)	AE	Severity	Disease duration	History or presence of AID	ASST	Inflammation markers			EC markers		
							CRP	ESR	Anemia	VEGF	SCF	sE‐selectin
Capillary density	0.94	0.45	0.08	0.50	0.87	0.72	0.41	0.64	0.23	0.30	0.79	0.49
Normal cap	0.95	0.81	0.11	0.75	0.97	0.91	0.13	0.34	**0.044**	**0.035**	0.53	0.56
Total abnormal cap	0.71	0.28	0.92	0.48	0.66	0.33	0.10	0.26	0.054	**0.019**	0.44	0.90
%Abnormal/total nb cap	0.79	0.33	0.79	0.51	0.63	0.51	0.059	0.21	**0.038**	**0.004**	0.48	0.89
Ramified cap	0.80	0.59	0.29	0.86	**0.047**	0.63	0.52	0.21	0.059	0.39	0.69	0.55
%Ramified/total nb cap	1.00	0.70	0.17	0.90	**0.005**	0.55	0.29	0.58	0.50	0.80	0.47	0.63
Concave cap	0.40	0.80	0.76	0.38	0.41	0.72	0.35	0.50	0.75	0.25	0.96	0.80
%Concave/total nb cap	0.58	0.92	0.24	0.07	0.39	0.59	0.86	0.32	0.49	0.10	0.83	0.91
Crossing cap	1.00	0.30	0.64	0.19	0.33	0.36	0.12	0.60	0.15	0.019	0.37	0.81
%Crossing/total nb cap	0.63	0.85	0.92	0.11	0.18	0.91	0.38	0.69	0.92	0.13	0.79	0.75
Regularly enlarged cap	0.41	0.83	0.47	0.67	**0.049**	0.71	0.24	0.12	0.66	0.07	0.84	0.07
Irregularly enlarged cap	0.80	0.52	0.47	0.82	0.31	0.71	0.47	0.14	0.96	0.74	0.47	**0.043**
Giant cap	ND	0.46	ND	ND	0.69	0.19	ND	ND	ND	ND	ND	ND
total nb of dilated cap	0.51	1.00	0.67	0.67	**0.048**	0.68	0.23	0.08	0.70	0.15	0.97	**0.032**
%nb dil/total nb cap	0.50	0.93	0.53	0.96	0.07	0.73	0.24	0.10	0.84	0.18	0.92	0.055
Microhemorrhages	0.19	0.81	0.70	0.76	0.22	0.79	0.51	0.49	0.65	0.95	0.21	0.42
NVC plexus score	0.95	0.72	0.97	0.39	0.67	0.49	0.16	0.58	0.64	0.75	0.65	0.88

*Note*: NVC parameters and their clinico‐serological associations are given using multiple regressions analyses, including age as a covariate and sex as a factor. *p*‐values are considered statistically significant when *p* < 0.05 (bold). Standard definitions of capillaroscopic evaluations suggested by the European League Against Rheumatism study group on microcirculation in rheumatic diseases (EULAR SG MC/RD) were applied^7^, ^38^. Capillary crossings were considered abnormal when >2 crossings were observed. The total number of abnormal shapes corresponded with the sum of all capillaries with multiple crossings (>2), ramifications and concave tips.

Abbreviations: AE, angioedema; AID, auto‐immune diseases; ASST, autologous serum skin test; cap, capillaries; CRP, C reactive protein; CSU, chronic spontaneous urticaria; EC, endothelial cell; ESR, erythrocyte sedimentation rate; nb, number; NS, not significant; NVC, nailfold videocapillaroscopy; SCF, stem cell factor; VEGF, vascular endothelial growth factor.

In CSU, sE‐selectin levels were linked to a history of AID (65.17 ± 6.07 pg/mL, *p* = 0.002) but not to low IgE and/or high anti‐TPO levels (*p* = 0.278). VEGF and SCF levels in patients with CSU were not linked to a history/presence of AID (*p* = 0.28 and *p* = 0.81, respectively) or low IgE and/or high anti‐TPO levels (*p* = 0.299 and *p* = 0.832, respectively).

## 4 DISCUSSION

This first NVC study in CSU identified specific nailfold capillary abnormalities that could imply systemic vascular involvement in the pathogenesis of CSU.

Our findings that CSU patients have significantly lower capillary density and more capillary malformations and irregular dilations compared with HCs are reminiscent of the results of a recent NVC study in hereditary AE by Cesoni et al.^40^ In this study, patients with hereditary AE with C1‐inhibitor deficiency (HAE‐C1INH) also had decreased capillary density and increased capillary dilations with more tortuous and ramified capillaries. HAE‐CINH and CSU are different in their pathomechanisms, but share some similarities in their clinical manifestations, with recurrent AE; additionally, their lesions are both driven by vascular activation and increased extravasation. The similarities in NVC signatures in patients with CSU in our study and those with HAE‐C1INH suggest that recurrent episodic activation of ECs, independent of the activating mechanism, can result in systemic microcirculatory changes. Hypothetically, the increase in abnormally shaped capillaries in CSU could imply the systemic presence of pro‐angiogenic signals, with later progression to a loss of ECs, resulting in reduced capillary density. To further attest to this, longitudinal NVC‐studies in CSU are warranted.

Interestingly, CSU signature changes of nailfold capillary density, dimensions and morphology were less pronounced in patients with a history/presence of AID and in those with low IgE levels and/or increased anti‐TPO levels, which are markers of type IIb aiCSU.^41^, ^42^ This is unexpected since aiCSU is usually characterized by high disease activity, resistance to standard treatments, and a high rate of AE.^43^, ^44^ At present, we cannot explain why patients with markers of aiCSU show less pronounced capillary abnormalities compared with patients without these markers. In previous studies performed at specialist urticaria centers, patients with markers of aiCSU had a shorter duration of disease, that is, time from the onset of CSU to inclusion in the study. This may be due to the higher disease activity and resistance to standard treatment of aiCSU, driving these patients to seek expert help faster than patients with non‐aiCSU. In our study, which was also done at a specialist urticaria center, patients with markers of aiCSU did not have significantly different disease durations nor disease activity compared with patients without markers of aiCSU. In any case, our results suggest that NVC could be a useful clinical tool for the identification of aiCSU patients.

We assessed possible mechanisms of microcirculatory changes on NVC in patients with CSU by looking at certain mediators, that is, sE‐selectin, SCF and VEGF, all of which are known to activate ECs and regulate vascular permeability. Although many symptoms of urticaria are mediated primarily by the actions of histamine on H1 receptors on ECs (resulting in wheals),^12^ other histamine‐independent mechanisms appear to contribute to vascular leakage, with direct or indirect influence on VEGF signaling.^11^ In CSU skin, VEGF is a potential marker for angiogenesis^17^, ^25^; nevertheless, for CSU serum clear evidence is still lacking.^17^, ^19^, ^20^ Although there is increasing evidence that VEGF is overexpressed in different conditions including chronic inflammation, we were unable to demonstrate significantly upregulated VEGF levels in the blood of patients with CSU compared to HCs. Nevertheless, patients with CSU with increased VEGF levels had significantly more abnormal capillaries and, more crossing capillaries compared with those with lower VEGF levels. This correlation has also been shown in patients with systemic lupus erythematosus.^45^, ^46^, ^47^, ^48^, ^49^, ^50^ For systemic sclerosis or HAE‐C1INH, no NVC correlations with VEGF have been found.^40^, ^51^, ^52^ Although several authors show upregulation of E‐selectin in (lesional and non‐lesional) skin of patients with CSU,^53^, ^54^, ^55^, ^56^, ^57^ research on sE‐selectin serum levels is controversial^55^, ^58^ and lacking.^11^ In our study, sE‐selectin serum levels were not significantly elevated compared with HCs. Furthermore, we were not able to show elevated serum SCF levels in CSU, which is in line with earlier studies of serum SCF in CSU and chronic inducible urticaria.^30^

Our study has several strengths. We performed comprehensive nailfold capillary analyses by NVC, a golden standard approach for the identification and characterization of systemic microcirculatory changes. Moreover, our patients were well characterized clinically and in terms of laboratory markers. Three possible molecular EC markers of microcirculatory changes were observed in combination with NVC assessment. Our NVC findings underline the potential microvascular impact of the chronic nature of CSU and offer perspectives for new research on why CSU has a long disease duration, recurs frequently after years or sometimes comes with aspecific systemic clinical features. Limited data on associations with hypertension or metabolic syndrome have been published in the past.^59^, ^60^, ^61^, ^62^ Although the NVC changes found in our study seemingly remain subclinical in patients with CSU, future studies should focus more on the detection and origin of long term systemic (vascular) complaints and comorbidities.

As for limitations, firstly, our study was monocentric, and the number and diversity of our patients were limited, for NVC as well as for ELISA. Also, there is a lack of information on aiCSU‐defining outcomes such as MC‐activating autoantibodies and basophil testing in our study that does not allow for definite conclusions. Therefore, further studies are needed and should be performed across several centers with larger and more diverse patient populations. The global network of urticaria centers of reference and excellence appears ideally suited to perform such studies.^63^ Secondly, though NVC is the golden standard method for detecting microvasculopathy in rheumatic diseases, its validity in inflammatory skin diseases remains poorly investigated challenging the interpretation of our results. Since NVC is readily available, straightforward, and inexpensive, further application of NVC in future CSU studies should be combined with clinical and serological assessments.

## 5 CONCLUSION

Taken together, our study identifies abnormalities of the microcirculation in CSU patients and provides further evidence that CSU has systemic effects beyond the development of skin lesions. Circulating VEGF and sE‐selectin appear to be linked, at least in part, but these findings need to be confirmed and extended to fully understand the underlying mechanisms. To this end, it would be interesting for future studies to focus on other EC markers reflecting EC damage such as endostatin, endoglin, angiopoietin, microparticles, and anti‐EC antibodies. From a clinical perspective, it will be important to show if effective treatment can reverse the systemic vascular involvement in patients with CSU, and if early treatment can prevent it.

## AUTHOR CONTRIBUTIONS

**Yora Mostmans**: substantial contributions to design of the study, acquisition of data and analysis and interpretation of data, drafting of the article, final approval of the version to be published and agreed to be accountable for all aspects of the work in ensuring that questions related to the accuracy or integrity of any part of the work are appropriately investigated and resolved. **Marcus Maurer**: interpretation of data, drafting of the article, critical revising of the article for important intellectual content and final approval of the version to be published and agreed to be accountable for all aspects of the work in ensuring that questions related to the accuracy or integrity of any part of the work are appropriately investigated and resolved. **Bertrand Richert**: contributions to design of the study, interpretation of data, critical revising of the article for important intellectual content and final approval of the version to be published and agreed to be accountable for all aspects of the work in ensuring that questions related to the accuracy or integrity of any part of the work are appropriately investigated and resolved. **Vanessa Smith**: contributions to design of the study, acquisition and interpretation of data, critical revising of the article for important intellectual content and final approval of the version to be published and agreed to be accountable for all aspects of the work in ensuring that questions related to the accuracy or integrity of any part of the work are appropriately investigated and resolved. **Karin Melsens**: acquisition and interpretation of data, critical revising of the article for important intellectual content and final approval of the version to be published and agreed to be accountable for all aspects of the work in ensuring that questions related to the accuracy or integrity of any part of the work are appropriately investigated and resolved. **Viviane De Maertelaer**: analysis and interpretation of data, critical revising of the article for important intellectual content and final approval of the version to be published and agreed to be accountable for all aspects of the work in ensuring that questions related to the accuracy or integrity of any part of the work are appropriately investigated and resolved. **Ines Saidi**: acquisition and interpretation of data, critical revising of the article for important intellectual content, final approval of the version to be published and agreed to be accountable for all aspects of the work in ensuring that questions related to the accuracy or integrity of any part of the work are appropriately investigated and resolved. **Francis Corazza**: acquisition of data, analysis and interpretation of data, critical revising of the article for important intellectual content, final approval of the version to be published and agreed to be accountable for all aspects of the work in ensuring that questions related to the accuracy or integrity of any part of the work are appropriately investigated and resolved. **Olivier Michel**: substantial contributions to design of the study, acquisition of data, analysis and interpretation of data, drafting of the article, critical revising of the article for important intellectual content, final approval of the version to be published and agreed to be accountable for all aspects of the work in ensuring that questions related to the accuracy or integrity of any part of the work are appropriately investigated and resolved.

## CONFLICT OF INTEREST STATEMENT

Yora Mostmans: reports a grant from The Brugmann Foundation for scientific research in chronic urticaria. Marcus Maurer: declares no conflict of interest in relation to this work. Outside of it, Marcus Maurer is or recently was a speaker and/or advisor for and/or has received research funding from Allakos, Alvotech, Amgen, Aquestive, Aralez, AstraZeneca, Bayer, Celldex, Celltrion, Evommune, GSK, Ipsen, Kyowa Kirin, Leo Pharma, Lilly, Menarini, Mitsubishi Tanabe Pharma, Moxie, Noucor, Novartis, Orion Biotechnology, Resoncance Medicine, Sanofi/Regeneron, Septerna, Trial Form Support International AB, Third HarmonicBio, ValenzaBio, Yuhan Corporation, and Zurabio. Bertrand Richert: declares no conflict of interest in relation to this work. Vanessa Smith: is a Senior Clinical Investigator of the Research Foundation ‐ Flanders (Belgium) (FWO) [1.8.029.20N]. Vanessa Smith is supported by an unrestricted educational chair on systemic sclerosis of Janssen‐Cilag NV. Vanessa Smith has received grant/research support from the Belgian Fund for Scientific Research in Rheumatic Diseases (FWRO) and Boehringer‐Ingelheim Pharma GmbH&Co; consulting fees were provided by Boehringer‐Ingelheim GmbH&Co and Janssen‐Cilag NV; speaker fees were provided by UCB Biopharma Sprl, Boehringer‐Ingelheim GmbH&Co, Janssen‐Cilag NV and Actelion Pharmaceuticals. Karin Melsens: declares no conflict of interest in relation to this work. Viviane De Maertelaer: declares no conflict of interest in relation to this work. Ines Saidi: declares no conflict of interest in relation to this work. Francis Corazza: declares no conflict of interest in relation to this work. Olivier Michel: declares no conflict of interest in relation to this work.

## Data Availability

The data that support the findings of this study are available from the corresponding author upon reasonable request.
